# Fluid Status in Peritoneal Dialysis Patients: The European Body Composition Monitoring (EuroBCM) Study Cohort

**DOI:** 10.1371/journal.pone.0017148

**Published:** 2011-02-24

**Authors:** Wim Van Biesen, John D. Williams, Adrian C. Covic, Stanley Fan, Kathleen Claes, Monika Lichodziejewska-Niemierko, Christian Verger, Jurg Steiger, Volker Schoder, Peter Wabel, Adelheid Gauly, Rainer Himmele, on behalf of the EuroBCM study group

**Affiliations:** 1 University Hospital Ghent, Ghent, Belgium; 2 University Hospital of Wales College of Medicine, Cardiff, United Kingdom; 3 University “Gr T Popa” and University Hospital “C I Pharon”, Iasi, Romania; 4 The Royal London Hospital, London, United Kingdom; 5 University Hospital Leuven, Leuven, Belgium; 6 Dialysis Center NephroCare, Gdansk University, Gdansk, Poland; 7 University Hospital René Dubos, Pontoise, France; 8 University Hospital Basel, Basel, Switzerland; 9 Fresenius Medical Care Deutschland GmbH, Bad Homburg, Germany; L' Istituto di Biomedicina ed Immunologia Molecolare, Consiglio Nazionale delle Ricerche, Italy

## Abstract

**Background:**

Euvolemia is an important adequacy parameter in peritoneal dialysis (PD) patients. However, accurate tools to evaluate volume status in clinical practice and data on volume status in PD patients as compared to healthy population, and the associated factors, have not been available so far.

**Methods:**

We used a bio-impedance spectroscopy device, the Body Composition Monitor (BCM) to assess volume status in a cross-sectional cohort of prevalent PD patients in different European countries. The results were compared to an age and gender matched healthy population.

**Results:**

Only 40% out of 639 patients from 28 centres in 6 countries were normovolemic. Severe fluid overload was present in 25.2%. There was a wide scatter in the relation between blood pressure and volume status. In a multivariate analysis in the subgroup of patients from countries with unrestricted availability of all PD modalities and fluid types, older age, male gender, lower serum albumin, lower BMI, diabetes, higher systolic blood pressure, and use of at least one exchange per day with the highest hypertonic glucose were associated with higher relative tissue hydration. Neither urinary output nor ultrafiltration, PD fluid type or PD modality were retained in the model (total R^2^ of the model = 0.57).

**Conclusions:**

The EuroBCM study demonstrates some interesting issues regarding volume status in PD. As in HD patients, hypervolemia is a frequent condition in PD patients and blood pressure can be a misleading clinical tool to evaluate volume status. To monitor fluid balance, not only fluid output but also dietary input should be considered. Close monitoring of volume status, a correct dialysis prescription adapted to the needs of the patient and dietary measures seem to be warranted to avoid hypervolemia.

## Introduction

Euvolemia is a predictor of outcome in peritoneal dialysis (PD) patients, as [1], [2]volume overload is related to cardiac dysfunction [3], [4], [5], inflammation [6] and mortality [7]. Euvolemia is probably a more important adequacy parameter than small solute clearance, as fluid status [7] but not small solute clearance [8] predicts outcome. Guidance on how to achieve and maintain euvolemia in individual PD patients is hampered by the absence of a convenient device to measure volume status, and by the lack of insight in the prevalence of and factors associated with volume overload.

In clinical practice, the assessment of volume status is relatively crude. Volume status is often assessed indirectly by measuring fluid removal, failing to take into account fluid balance by omission of dietary fluid intake. Ultrasonic evaluation of inferior vena cava diameter (IVC) only assesses intravascular volume, and is also influenced by diastolic dysfunction [9] [10], and is thus a reflection of preload, and not of tissue hydration[11]. Parameters, such as Brain Natriuretic Peptide (BNP) or NT-proBNP can reflect changes in hydration status [12], but are also influenced both by preload and ventricular abnormalities, and in patients with renal failure, accumulation can occur [13]. Direct measurement of extracellular (ECW) and total body water (TBW) by dilution methods is considered as the golden standard, but these techniques are laborious and expensive [14].

Bio-impedance spectroscopy (BIS) represents a different approach to the assessment of fluid status [11], [15], [16]. By measuring the flow of electrical current through the body, resistance and reactance can be measured, and in BIS, this is performed at different frequencies [17]. The Body Composition Monitor (BCM, Fresenius Medical Care, Germany) is a bio-impedance spectroscopy device for clinical use, validated by isotope dilution methods [18], and reference body composition methods [19], and has been used in hemodialysis (HD) [20], [21], [22], [23] and PD [24].

The fluid status in PD patients has so far not been characterized by a method that allows comparison to the normal healthy populations. Some studies have evaluated the volume status of PD patients in relation to modality (APD vs. CAPD) [25], [26] transport status, residual renal function [27], or inflammation [28]. However, whereas these studies contribute information on relative volume status in different groups of PD, they were hampered to express the degree of true fluid overload due to the lack of a reference population. In contrast, Wieskotten et al [29] evaluated a large cohort of 688 healthy persons using the BCM to derive reference ranges, allowing to compare fluid overload as measured by BCM to age and gender matched values of the normal healthy population. In addition, expressing extracellular and intracellular water as absolute values induces the problem of scaling to body size. In previous studies using bio-impedance, ratios of extracellular water to height, weight, body surface area, intracellular water or total body water [30] have been used to express “fluid overload”, but the ideal scaling parameter remains a matter of debate [14]. The use of relative Δtissue hydrationdiminishes the problem of scaling nearly completely, and allows comparison to the healthy population [29]. In HD patients [20] relative Δtissue hydration is associated with mortality, indicating the clinical relevance of this parameter.

The European Body Composition study (EuroBCM study) in PD was designed to measure hydration status in a large, multicentric cohort of PD patients using the BCM device, as compared to a healthy reference population, and to establish associations between clinical and practice related parameters and volume status.

## Methods

### Study objectives

The EuroBCM study in PD was a cross sectional, observational, multi center trial in 28 centers in 6 European countries. The primary objective was to analyze hydration status in a representative sample of prevalent PD patients as compared to the healthy population, and to identify associations between hydration status and patient characteristics (age, gender, diabetes, peritoneal transport characteristics, residual renal function, and daily ultrafiltration) and treatment practice (type of PD solution, use of APD vs CAPD) to find out which conditions should alert the clinician to potential fluid overload.

### Centers

Patients were recruited from 6 different European countries (Belgium, France, Poland, Romania, United Kingdom, and Switzerland). Centers were selected to reflect the distribution of PD in that country, aiming to an overall inclusion of ±10% of the total number of PD patients of that country.

### Patients

In each center, all prevalent patients on PD were assessed for eligibility for inclusion (prevalent cross-sectional cohort approach) if they were older than 18 years of age and wanted to sign informed consent. Patients were excluded if they had a cardiac pacemaker or metallic implants, were amputees or were pregnant. Patients were evaluated during a routine clinical visit. All patients signed informed consent, and ethical advice was obtained from the individual ethics committees as per country protocol. This trial has been registered at the Cochrane Renal Group trials registry (http://www.cochrane-renal.org) under the number CRG110800153.

### Measurements of hydration and body composition

BCM measurements were in each center performed by one reference PD physician or nurse, using a portable whole body bio-impedance spectroscopy device, the BCM (Fresenius Medical Care). The BCM measures the impedance spectroscopy at 50 different frequencies between 5 kHz and 1 MHz. The BCM was validated intensively against all available gold-standard methods [19]. Clinically relevant parameters were registered in the case report form (CRF).

Electrodes were attached to one hand and one foot at the ipsilateral side, after the patient had been in recumbent position for at least 5 minutes. Due to bio-physical reasons, bio-impedance spectroscopy does not measure sequestered fluid in the trunk [25], [31], [32], [33]. Therefore, presence or absence of PD fluid in the abdomen does not influence the readings of hydration status. For determination of weight, we used the weight adjusted for empty abdomen.

Extracellular water (ECW), intracellular water (ICW) and total body water (TBW) were determined from the measured impedance data following the model of Moissl et al [18]. Reproducibility of BCM derived parameters is high, with a coefficient of variation for the interobserver variability ECW and TBW around 1.2% [34]. Therefore, only one BCM measurement was performed in each individual patient.

Absolute ΔTissue Hydration (AΔTH) was derived from the impedance data based on a physiologic tissue model [35], [36]. Absolute ΔTissue Hydration represents the difference between the amount of ECW in the tissue as actually detected by the BCM and the amount of water present in tissue, as predicted by physiological models under normal physiological (normohydrated) conditions [36]. Of note, AΔTH has no direct relation to circulating volume.

All values of AΔTH were compared with and categorized according to the 10^th^ (corresponding to −1.1l) and 90^th^ (corresponding to +1.1l) percentiles of a population of the same gender distribution and with a comparable age band out of a healthy reference cohort, where hydration status was measured with the identical technology [29], [37].

AΔTH is further normalized to extracellular water, and expressed as a ratio called Relative ΔTissue Hydration (RΔTH  = AΔTH/ECW). In the normal reference population, the 90^th^ percentile of RΔTH is 7%. Accordingly, when RΔTH was greater than 7%, this was classified as “fluid overload”. As a RΔTH ratio >15% is related to mortality [20], this cut off was used to define “severe fluid overload”.

Blood pressure was recorded as the mean of two consecutive measurements with 5 minutes interval, using one single calibrated device in each center. Height and weight were measured using one single calibrated device in each center.

### Patient characteristics

Diabetes was assumed to be present in patients using glucose lowering drugs or insulin.

Congestive heart failure was defined according to the New York Heart Association (NYHA) classification. Ultrafiltration was calculated from the patient's charts as a daily mean of ultrafiltration (in ml) obtained during the last month preceding the measurement. Due to the daily variation, residual diuresis was assessed in a categorical way (<100 ml, between 100 and 500 ml/day, between 500 and 1000 ml/day, or >1000 ml/day) based on the reported current urine production. Total fluid output was estimated as the sum of urinary, taken as the halfway value of the cohort, and ultrafiltered volume per 24 hour. In this way, a patient with zero ultrafiltration and a reported urinary output in the 500–1000 ml/day has a total output of 750 ml, the cut off value in the EAPOS study [38].

The following biochemical parameters were determined in the local laboratories from blood collected during the routine visit: hemoglobin, hematocrit, albumin, CRP, urea, creatinine.

Peritoneal membrane characteristics were determined based on results of the last available PET test preceding the BCM measurement, according to Twardowski [39]. If no PET test was available the last four months, transport status was noted as “unknown”

### Statistical analysis

Continuous data are expressed as mean ± standard deviation. Categorical variables are expressed as percentage of total. For univariate comparisons, student's t-test, Mann Whitney U-test and Fisher's exact test were used. One-way ANOVA was used to compare multiple categories, with post hoc testing.

Multivariate linear regression analysis was performed with relative Δtissue hydration as the target variable, to find factors which were independenly associated with overhydration, and should thus alert the physician for this condition. Switzerland was excluded from the multivariate analysis as the low patient number made the models unstable. Since the implementation of APD and polyglucose was very low in Romania and Poland, it was decided to analyse only patients from UK, Belgium and France in the multivariate analysis.

Variables were selected for entry in the model selection procedure either because of univariate p<0.1 or for biological plausibility. Regression diagnostics was performed to detect and eliminate outliers and highly influential observations.

All analyses were done with SAS V9.2 (SAS Institute inc, Cary, North Carolina).

## Results

Of the prevalent patients in the study centers, 734 were eligible for the study, 73 of whom were excluded because of predefined contra-indications for BCM measurement: metal implants or artificial joints: n = 48, pacemakers or implanted pumps: n = 15, amputations: n = 10. From the remaining 661 patients, 22 patients had incomplete data.

Patients were recruited from Belgium (5 centers, n = 98), France (5 centers, n = 65), Poland (5 centers, n = 82), Romania (9 centers, n = 218), United Kingdom (2 centers, n = 167) and Switzerland (1 center, n = 9).

The baseline demographic, clinical, relevant laboratory data and hydration parameters of the population are provided in table 1. In this population, 24.4% were diabetic, and 32.1% had signs of heart failure (9.7, 12.2, 8.1 and 2.0% NYHA class 1, 2, 3 or 4 respectively). Some patients had previously been treated by HD (18.3%), or had a failed transplant (4.9%). Average time on PD was 32.6±31.0 months. At least one type of antihypertensive drug was taken by 85.4% of the patients (44.9% diuretics, 46.8% Beta blocking agents, 41.5% calcium antagonists, 51.2% inhibitors of the renin- angiotensin system, 9% central acting drugs).

**Table 1:** Demographic, clinical and fluid status data of the EuroBCM study cohort (N = 639).

	mean or percentage	Standard deviation
**Gender Male**	55%	
**Age (years)**	58.8	14.8
**Height (cm)**	165.7	9.6
**Weight (kg)**	72.2	15.4
**Body Mass Index (kg/m^2^)**	26.3	5.1
**Blood pressure (mmHg)** **Systolic** **Diastolic**	136.979.9	25.614.3
**Residual GFR (ml/min)**	6.6	7.2
**Ultrafiltration (ml/day)**	940	580
**Residual urine output** **<100 ml/day** **100–500 ml/day** **500–1000 ml/day** **>1000 ml/day** **Missing data**	19.1%21.9%23.5%32.6%3.0%	
**Treatment modality Automated PD** §	53.1%	
**Use of polyglucose** §	63.7%	
**Transport status** **Fast** **Fast average** **Slow average** **Slow** **Not known**	16.6%33.3%28.3%5.9%15.9%	
**Serum levels** **Albumin (g/l)** **Creatinine (mg/dl)** **Urea (mg/dl)** **C-reactiveprotein (mg/l)** **Hemoglobin (g/dl)** **Hematocrit (%)** **HbA1C (%)**	36.38.1117.011.611.334.36.5	6.03.039.723.51.65.11.7
**Absolute** Δ**Tissue Hydration (A**Δ**TH) (l)**	1.7Q25: 0.2; Median 1.3; Q75: 2.9	2.3
**Relative** Δ**Tissue Hydration (%) (Ratio A**Δ**TH/ECW)**	8.6Q25: 1.1; Median 7.8; Q75: 15.1	11.5
**Total Body Water (l)**	35.8	7.7
**Extracellular Water (l)**	17.2	3.8
**Intracellular water (l)**	18.5	4.5
**Extracellular/Intracellular water**	0.95	0.15
**Intracellular resistance Ri (Ohm/m)**	569.6	117.5
**Extracellular resistance Re (Ohm/m)**	1611.6	479.5
**Phase angle at 50 kHz**	4.9	1.2

§ after exclusion of patients from countries where polyglucose and APD are not liberally available due to logistical reasons.

Underhydration (AΔTH<10^th^ percentile), normohydration and overhydration (AΔTH>90^th^ percentile), as defined by the 10^th^ and 90^th^ percentile of values obtained in the normal population [29], were present in 6.7, 39.9 and 53.4% of the EuroBCM cohort. Fluid overload and severe fluid overload, as defined by a relative Δtissue hydration (AΔTH/ECW) above 7% or above 15% were present in 53.4 and 25.2% of the study population.

### Univariate analysis

**Figure 1:**
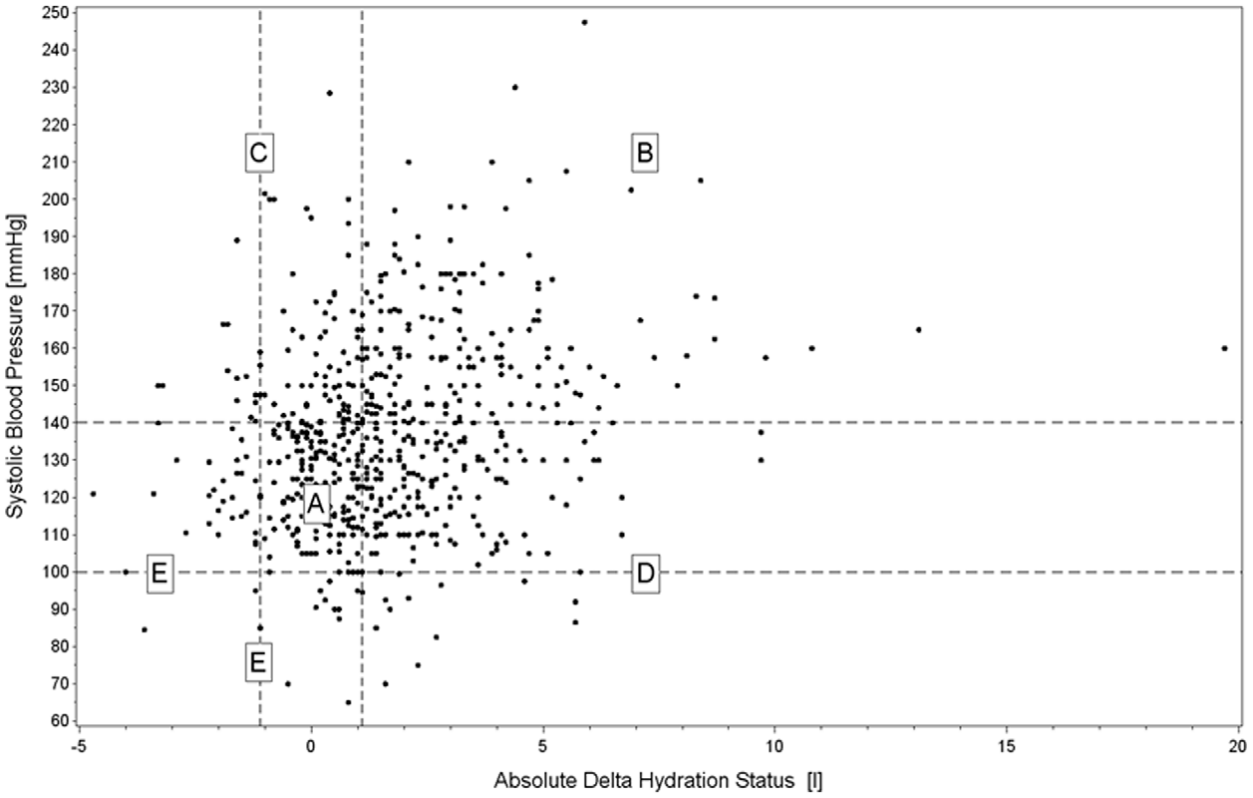
Scatter plot of the relation between absolute Δtissue hydration (litres) in the X-axis and systolic blood pressure (mmHg) in the Y-axis in the individual patients of the EuroBCM study cohort. Dotted vertical lines indicate the 10^th^ and 90^th^ percentile of absolute Δtissue hydration in the healthy population (−1.1 and +1.1 liter respectively), representing thus the limits of “normohydration”. Dotted horizontal lines indicate the “normotensive range” for systolic blood pressure.

There was a substantial scatter on the linear relationship between AΔTH and systolic blood pressure, diastolic blood pressure or pulse pressure (correlation coefficients 0.23, 0.02 and 0.29 respectively). As described elsewhere for HD patients [22], [37], different zones (figure 1) can be identified in the plot of systolic blood pressure (Y-axis) versus AΔTH (X-axis), of patients who are both normohydrated and normotensive (26.8%, zone A), who are both fluid overloaded and hypertensive (25.8%, zone B), who are hypertensive despite being normo- or underhydrated (13.3%, zone C), who are normo- and hypotensive despite being fluid overloaded (27.5%, zone D) and patients who are hypotensive and normohydrated or normotensive and underhydrated (6.6%, zone E)

Males (vs females, 2.19±2.57 vs 1.03±1.82 l, p<0.001) and diabetics (vs non diabetics, 1.92±2.12 vs 1.52±2.38 l, p = 0.06) had a higher AΔTH. The prevalence of PD patients with a AΔTH>90^th^ percentile of the normal healthy reference population was also higher in males as compared to females (65.0 vs 39.3%). There was no impact of “vintage on PD” on AΔTH (time on PD of patients with an AΔTH >1.1 liter vs euvolaemic patients 32.5±28.0 vs 33.4±34.4 months, p = 0.66).

**Figure 2:**
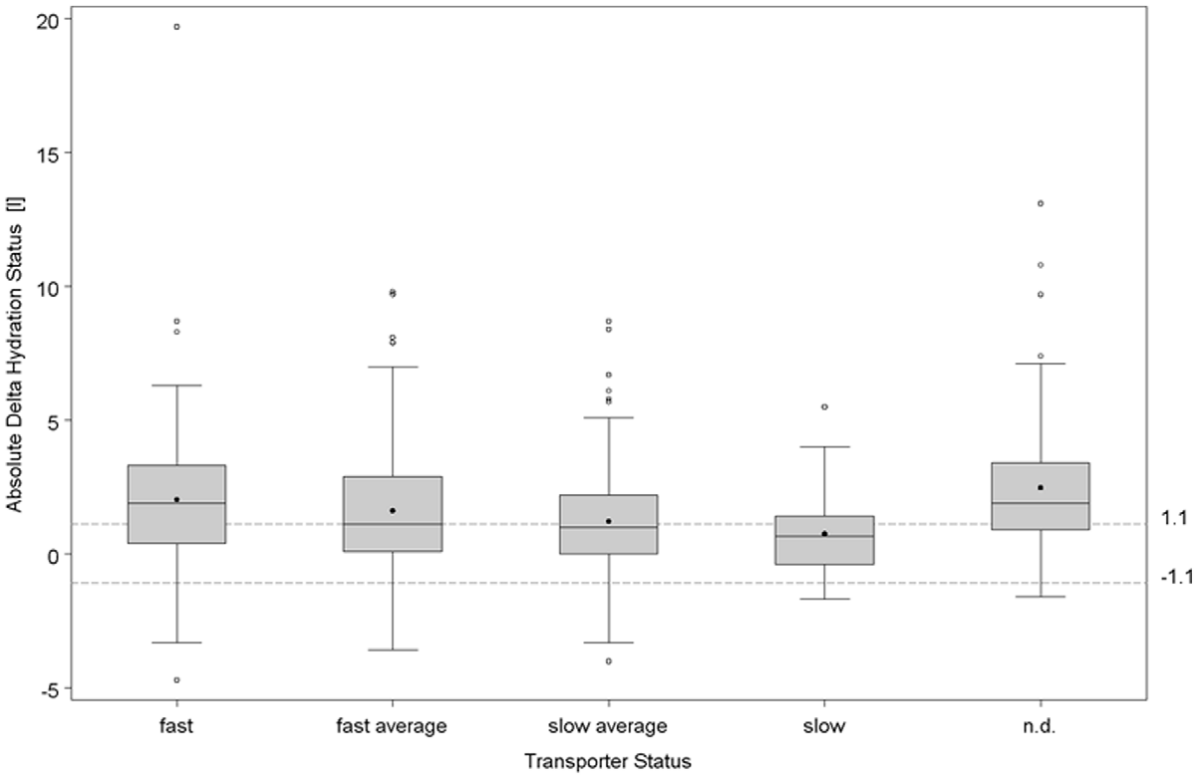
Box and whisker plots (median, 25^th^ and 75^th^ quartile, outliers) of Absolute ΔTissue Hydration (in liters) in the different transport categories. n.d.: no peritoneal transport characteristics available in the 4 months before the BCM measurement.

There was a correlation between transport status and AΔTH (figure 2), with a declining trend from fast (2.04±2.75 l) to fast average (1.63±2.34 l), slow average (1.23±1.97 l) and slow (0.76±1.71 l) transport status (ANOVA: p<0.001). However the interquartile range in each group was substantial, and there is considerable overlap in AΔTH between the groups. AΔTH was most increased in those patients where transport status had not routinely been measured in the last four months (2.48±2.42 l, post-hoc p-value vs. slow transport status p<0.0001). There was a trend for declining AΔTH with increasing urinary output from <100 ml/day (1.99±2.38 l), over 100–500 ml/day (1.84±2.77 l) and 500–1000 ml/day (1.55±2.12 l) to those with a urinary output greater than 1000 ml/day (1.28±1.99 l) (one way ANOVA: p<0.001), but with large interquartile range and overlap. There was no correlation between AΔTH and daily ultrafiltration (R = 0.10), and only a weak correlation between AΔTH and estimated daily total fluid output (residual diuresis + peritoneal ultrafiltration) (R = 0.17).

There was a negative relation between AΔTH and serum albumin (R = −0.42), hemoglobin (R = −0.34) and hematocrit (R = −0.31). There was no correlation between AΔTH and glomerular filtration rate or CRP-level.

There was a difference in AΔTH in the univariate analysis between patients using or not using polyglucose (0.9±2 vs 1.4±2 l resp, p = 0.04) in the countries without logistical impediment to the use of polyglucose. The relation between hydration status and use of polyglucose was complex, with more patients being overhydrated in the group using polyglucose in Belgium, whereas in UK and France, patients using polyglucose were less overhydrated (table 2). In countries where the use of polyglucose was restricted (Romania and Poland), the few patients using polyglucose tended to be more overhydrated (table 2), potentially indicating a bias by indication.

**Table 2:** Tissue hydration related to percentiles of the normal reference population stratified for the use of polyglucose or not.

	% <10^th^ percentile of normal population	% between 10^th^ and 90^th^ percentile of normal population	% >90^th^ percentile of normal population
BelgiumPolyglucose (n = 59)No polyglucose (n = 39)	6.85.1	42.456.4	50.838.5
FrancePolyglucose (n = 44)No polyglucose (n = 21)	9.14.8	54.533.3	36.461.9
United KingdomPolyglucose (n = 113)No polyglucose (n = 54)	16.89.3	47.838.9	35.451.8
SwitzerlandPolyglucose (n = 7)No polyglucose (n = 2)	0.00.0	0.050.0	100.050.0
RomaniaPolyglucose (n = 17)No polyglucose (n = 203)	1.02.8	33.035.3	66.060.0
PolandPolyglucose(n = 9)No polyglucose (n = 73)	4.111.1	35.644.4	60.344.4

There was a small difference in AΔTH in univariate analysis between patients on CAPD vs APD (1.3±2.0 vs 0.9±1.9 l resp, p = 0.06) in these countries without logistical impediment to the use of APD (Belgium, France, UK).

### Multivariate analysis of tissue hydration

Because of the strong interaction, the multivariate analysis included only patients from countries with unrestricted access to APD and alternative PD solutions.

In this multivariable linear regression analysis adjusted for country effects (table 3), older age, male gender, lower serum albumin, lower BMI, diabetes, higher systolic blood pressure, and use of at least once per day highest hypertonic glucose were associated with higher relative tissue hydration. Neither urinary output nor ultrafiltration was retained in the model. The use of alternative dialysis solutions (including polyglucose) did not contribute to the model (total R^2^ of the model = 0.57).

**Table 3:** Multivariate linear regression for Relative ΔTissue Hydration from the subgroup of patients from Belgium, France and UK.

Parameter	Coefficient	95% CI		p-value
Intercept	**30.27**	20.65	39.88	<0.0001
Age (per year)	**0.10**	0.05	0,16	0.0002
Sex (female vs male)	**−3.04**	−4.55	−1.52	0.0001
Albumin per g/l	**−0.75**	−0.91	−0.59	<0.0001
BMI per kg/m^2^	**−0.66**	−0.83	−0.50	<0.0001
Diabetes (vs no diabetes)	**4.86**	3.14	6.59	<0.0001
Systolic BP (per mmHg)	**0.09**	0.05	0.12	<0.0001
Glucose at least once 2.5% vs. 1.5% only	**−0.73**	−2.56	1.11	0.80
Glucose at least once 3.86/4.25% vs. 1.5% only	**5.18**	2.62	7.74	<0.0001

Not included due to p>0.1				
Ultrafiltration				0.86
Urine output				0.66
Hypertension stage				0.41
NYHA Stage				0.39
Liver disease				0.56
Time on PD per month				0.25
Transport status				0.83
Type of PD solution				0.12
PD modality				0.27

Model adjusted for country effects (Belgium, France and UK), total R^2^ of the model = 0.57, n = 299.

NYHA = New York Heart Association classification of heart failure.

## Discussion

The EuroBCM study is the first large multi-centre study of hydration status and its associated factors in PD patients in Europe allowing comparison to a healthy reference population. Fluid overload was a frequent finding in PD patients as compared to a healthy reference population [29], but comparable to that reported in HD patients [20], [22], [23]. The deviation from the relation between blood pressure and tissue hydration was substantial, pointing out that blood pressure is not a good tool to evaluate hydration status in PD patients. Overhydration was associated with higher age, male gender, diabetes, lower BMI, higher systolic blood pressure, and use of hypertonic solutions, and in these conditions, physicians should have enhanced awareness for volume status. Use of polyglucose or biocompatible glucose solutions or the type of PD modality was not independently associated with hydration status.

In the large cohort of the EuroBCM in PD study, a substantial portion of patients were fluid overloaded by more than 1.1 litre, the 90^th^ percentile of absolute Δtissue hydration in the normal reference population [29], and 25% of patients had a relativeΔ tissue hydration/extracellular water ratio above 15%, a value associated with increased mortality in HD patients [20]. Substantial fluid overload is therefore indeed a prevalent problem in PD patients, and more attention should be given to its assessment and correction. Nevertheless, it is important to note that comparable numbers of severe fluid overload have been reported in HD patients [20], [22], [23], [24], and already in early stages of renal impairment, patients tend to be more fluid overloaded [40], [41].

Many physicians estimate hydration status by using clinical parameters, such as edema, weight gain or blood pressure[42]. Although there was a direct correlation between systolic blood pressure and tissue hydration, a substantial proportion of patients did not comply with this paradigm. A number of patients had systolic hypertension, despite normohydration or even tissue underhydration. These are probably patients who suffer from vascular stiffness [2]. Further dehydration of these patients in an attempt to normalize blood pressure might be dangerous, as it might abruptly compromise coronary perfusion [43]. A number of patients had a low or normal blood pressure, despite being fluid overloaded. It is conceivable that many of these patients suffer congestive heart failure. Normotension in these patients should not be seen as equivalent to euvolemia, as also reported in HD patients [22].

In many studies on fluid overload, attention is focused on fluid output (ultrafiltration and/or diuresis), neglecting that fluid status is a balance of fluid output and input. In the EuroBCM study, there was a very weak association between fluid overload and diuresis, but this association disappeared in the multivariate analysis. Davison et al [25] found a small influence of residual GFR, but not of peritoneal ultrafiltration or daily urine output, on volume status. Wiggins et al [44] demonstrated that total fluid output one month after the initiation of PD was not associated with patient survival. All these point out that in studies on fluid status, both fluid input and output should be considered. In addition, and maybe even more of importance, clinicians should be aware that patients can be overhydrated because of dietary incompliance, despite having substantial residual diuresis. Dietary intake of fluid and salt should thus be conisdered when managing fluid overloaded patients.

In our BCM cohort, the use of high hypertonic bags was associated with fluid overload. It is tempting to attribute this observation to bias by indication. However, an alternative potential hypothesis could be that the strategy of using hypertonic bags is not effective in returning patients back to euvolemia for a sustained period of time, as it can lead to dysregulation of glycemic control, and thus to hyperosmolarity and thirst. Sustained exposure to hypertonic exchanges can also negatively impact on the peritoneal membrane function [45], leading to further detrimental consequences on fluid balance. Further studies in this regard are warranted. This is compatible with the negative impact of high initial peritoneal fluid removal [44]: it is likely that those with a high fluid output achieved this at the expense of increased use of hypertonic bags, thus damaging the peritoneal membrane in the long term.

There was an association between peritoneal membrane transport characteristics and tissue hydration, as already demonstrated by others [27]. Nevertheless, there was a substantial overlap between groups, and the effect was rather small and disappeared in the multivariate analysis. In the study by Davison et al [25], transport status explained only 1.6% of the variation in volume status. It can be hypothesized that fluid overload is induced by not adapting the dwell time appropriately to the transport status of the patient [46]. Although it has been stated that removal of salt can be impaired in patients on APD [47], hydration status in patients on APD and CAPD was comparable in the multivariate analysis in the EuroBCM cohort, just as in previous observations [25], [26]. Of note, in one of these studies [25], the number of cycles per night was limited, so the dwell time was probably long enough to allow diffusive sodium transport. To maintain fluid balance, fast transporters need short dwells, to avoid negative ultrafiltration, and implementing APD might be of value in this patient category. On the other hand, slow transporters need long dwells to avoid sodium sieving, and APD with short cycles might be detrimental in this patient group. Johnson et al [48] recently reported that APD was associated with better survival in fast, but with worse survival in low transporters, an observation that is compatible with this paradigm.

As Davison et al (23), we found a negative association between serum albumin and overhydration. As this is a cross-sectional cohort, it is however impossible to determine whether low albumin is a consequence or a cause of overhydration.

In the EuroBCM study cohort, polyglucose use was associated with less overhydration and more underhydration in some countries, whereas the opposite was true in other countries, pointing to potential underlying differences in practice related to the use of polyglucose (table 2). In a subcohort of the EuroBCM trial, excluding countries were alternative PD solutions and APD are not liberally available due to logistical reasons, we observed a neutral impact both of the solution type and the PD modality on fluid overload, just as it was found in the cohort of Davison et al [25].

This study is a cross sectional study, and as such, no causal relations can be drawn. However, our observations can generate some interesting hypotheses on the association between practices and hydration status. It would be interesting e.g. to study the impact on hydration status and residual renal function using a prospective protocol where implementation of polyglucose, dwell length and use of APD vs CAPD is guided by BCM based assessment of fluid overload. Another limitation is the rather crude evaluation of fluid output using patient charts as a reference, which might induce inaccuracies. However, this is the way fluid output is measured in real life. Of special interest for a future prospective study in this regard is the potential impact of bag overfill on the overestimation of ultrafiltration and fluid overload [49]. It can be that the overestimation of real ultrafiltration by neglecting overfill can lead to overhydration, as it gives the patient and the physician the false feeling of adequate ultrafiltration.

In conclusion, the EuroBCM study demonstrates some interesting issues on volume status in PD patients: fluid overload is a frequent problem, and relying only on clinical parameters for its assessment might be misleading. Fluid overload is related to prescription practices, gender and diabetes. Despite good ultrafiltration and residual diuresis, patients still can be fluid overloaded, stressing the important role of dietary restriction of salt and fluid intake. Although indication bias cannot be excluded, attempts to increase ultrafiltration by the long term use of hypertonic bags [46] seem to be no guarantee for achieving sustained euvolemia. Objective measurement of fluid status as a basis for an integrated approach to fluid balance is warranted. As fluid overload has been linked to mortality [7], [20], further studies evaluating whether awareness of hydration status can improve volume management and patient outcome are warranted.

## References

[1] Lo WK, Bargman JM, Burkart J, Krediet RT, Pollock C (2006). Guideline on targets for solute and fluid removal in adult patients on chronic peritoneal dialysis.. Perit Dial Int.

[2] Van Biesen W, Verbeke F, Devolder I, Vanholder R (2008). The relation between salt, volume, and hypertension: clinical evidence for forgotten but still valid basic physiology.. Perit Dial Int.

[3] Wang AY, Lam CW, Wang M, Chan IH, Goggins WB (2007). Prognostic value of cardiac troponin T is independent of inflammation, residual renal function, and cardiac hypertrophy and dysfunction in peritoneal dialysis patients.. Clin Chem.

[4] Konings CJ, Kooman JP, Schonck M, Dammers R, Cheriex E (2002). Fluid status, blood pressure, and cardiovascular abnormalities in patients on peritoneal dialysis.. Perit Dial Int.

[5] Enia G, Mallamaci F, Benedetto FA, Panuccio V, Parlongo S (2001). Long-term CAPD patients are volume expanded and display more severe left ventricular hypertrophy than haemodialysis patients.. Nephrol Dial Transplant.

[6] Demirci MS, Demirci C, Ozdogan O, Kircelli F, Akcicek F Relations between malnutrition-inflammation-atherosclerosis and volume status. The usefulness of bioimpedance analysis in peritoneal dialysis patients.. Nephrol Dial Transplant.

[7] Paniagua R, Ventura MD, Avila-Diaz M, Hinojosa-Heredia H, Mendez-Duran A (2010). NT-proBNP, Fluid volume overload and dialysis modality are independent predictors of mortality in ESRD patients.. Nephrol Dial Transplant.

[8] Paniagua R, Amato D, Correa-Rotter R, Ramos A, Vonesh EF (2000). Correlation between peritoneal equilibration test and dialysis adequacy and transport test, for peritoneal transport type characterization. Mexican Nephrology Collaborative Study Group.. Perit Dial Int.

[9] Leunissen KM, Kouw P, Kooman JP, Cheriex EC, deVries PM (1993). New techniques to determine fluid status in hemodialyzed patients.. Kidney Int Suppl.

[10] Aurigemma GP, Gaasch WH (2004). Clinical practice. Diastolic heart failure.. N Engl J Med.

[11] Kraemer M, Rode C, Wizemann V (2006). Detection limit of methods to assess fluid status changes in dialysis patients.. Kidney Int.

[12] Jacobs LH, van de Kerkhof JJ, Mingels AM, Passos VL, Kleijnen VW (2009). Inflammation, overhydration and cardiac biomarkers in haemodialysis patients: a longitudinal study.. Nephrol Dial Transplant.

[13] Sommerer C, Heckele S, Schwenger V, Katus HA, Giannitsis E (2007). Cardiac biomarkers are influenced by dialysis characteristics.. Clin Nephrol.

[14] Woodrow G (2007). Body composition analysis techniques in adult and pediatric patients: how reliable are they? How useful are they clinically?. Perit Dial Int.

[15] Matthie JR (2008). Bioimpedance measurements of human body composition: critical analysis and outlook.. Expert Rev Med Devices.

[16] Jaffrin MY, Morel H (2008). Body fluid volumes measurements by impedance: A review of bioimpedance spectroscopy (BIS) and bioimpedance analysis (BIA) methods.. Med Eng Phys.

[17] Kotanko P, Levin NW, Zhu F (2008). Current state of bioimpedance technologies in dialysis.. Nephrol Dial Transplant.

[18] Moissl UM, Wabel P, Chamney PW, Bosaeus I, Levin NW (2006). Body fluid volume determination via body composition spectroscopy in health and disease.. Physiol Meas.

[19] Wabel P, Chamney P, Moissl U, Jirka T (2009). Importance of whole-body bioimpedance spectroscopy for the management of fluid balance.. Blood Purif.

[20] Wizemann V, Wabel P, Chamney P, Zaluska W, Moissl U (2009). The mortality risk of overhydration in haemodialysis patients.. Nephrol Dial Transplant.

[21] Wizemann V, Rode C, Wabel P (2008). Whole-body spectroscopy (BCM) in the assessment of normovolemia in hemodialysis patients.. Contrib Nephrol.

[22] Passauer J, Petrov H, Schleser A, Leicht J, Pucalka K Evaluation of clinical dry weight assessment in haemodialysis patients using bioimpedance spectroscopy: a cross-sectional study.. Nephrol Dial Transplant.

[23] Machek P, Jirka T, Moissl U, Chamney P, Wabel P Guided optimization of fluid status in haemodialysis patients.. Nephrol Dial Transplant.

[24] Devolder I, Verleysen A, Vijt D, Vanholder R, Van Biesen W Body composition, hydration, and related parameters in hemodialysis versus peritoneal dialysis patients.. Perit Dial Int.

[25] Davison SN, Jhangri GS, Jindal K, Pannu N (2009). Comparison of volume overload with cycler-assisted versus continuous ambulatory peritoneal dialysis.. Clin J Am Soc Nephrol.

[26] Davenport A, Willicombe M (2009). Comparison of fluid status in patients treated by different modalities of peritoneal dialysis using multi-frequency bioimpedance.. Int J Artif Organs.

[27] Konings CJ, Kooman JP, Schonck M, Struijk DG, Gladziwa U (2003). Fluid status in CAPD patients is related to peritoneal transport and residual renal function: evidence from a longitudinal study.. Nephrol Dial Transplant.

[28] Gangji AS, Brimble KS, Margetts PJ (2009). Association between markers of inflammation, fibrosis and hypervolemia in peritoneal dialysis patients.. Blood Purif.

[29] Wieskotten S, Heinke S, Wabel P, Moissl U, Becker J (2008). Bioimpedance-based identification of malnutrition using fuzzy logic.. Physiol Meas.

[30] Engel B, Davies SJ (2007). Achieving euvolemia in peritoneal dialysis.. Perit Dial Int.

[31] Foster KR, Lukaski HC (1996). Whole-body impedance–what does it measure?. Am J Clin Nutr.

[32] Cooper BA, Aslani A, Ryan M, Zhu F, Ibels LS (2000). Comparing different methods of assessing body composition in end stage renal failure.. Kidney Int.

[33] Passauer J, Schewe J, Parmentier S, Palm C, Herbrig K (2009). Influence of peritoneal fluid on measurements of fluid overload by bio-impedance spectroscopy in peritoneal dialysis patients.. World Congres of Nephrology.

[34] Wabel P, Chamney P, Moissl U (2007). Reproducibility of bioimpedance spectroscopy for the assessment of body composition and dry weight.. J Am Soc Nephrol.

[35] Chamney PW, Wabel P, Moissl UM, Muller MJ, Bosy-Westphal A (2007). A whole-body model to distinguish excess fluid from the hydration of major body tissues.. Am J Clin Nutr.

[36] Wang J, Pierson R (1976). Disparate hydration of adipose and lean tissue require a new model for body water distribution in man.. J Nutr.

[37] Wabel P, Moissl U, Chamney P, Jirka T, Machek P (2008). Towards improved cardiovascular management: the necessity of combining blood pressure and fluid overload.. Nephrol Dial Transplant.

[38] Brown EA, Davies SJ, Rutherford P, Meeus F, Borras M (2003). Survival of functionally anuric patients on automated peritoneal dialysis: the European APD Outcome Study.. J Am Soc Nephrol.

[39] Twardowski Z, Nolph K, Khanna R (1987). Peritoneal equilibration test.. Perit Dial Bull.

[40] Bellizzi V, Scalfi L, Terracciano V, De Nicola L, Minutolo R (2006). Early changes in bioelectrical estimates of body composition in chronic kidney disease.. J Am Soc Nephrol.

[41] Essig M, Escoubet B, de Zuttere D, Blanchet F, Arnoult F (2008). Cardiovascular remodelling and extracellular fluid excess in early stages of chronic kidney disease.. Nephrol Dial Transplant.

[42] Cocchi R, Degli Esposti E, Fabbri A, Lucatello A, Sturani A (1999). Prevalence of hypertension in patients on peritoneal dialysis: results of an Italian multicentre study.. Nephrol Dial Transplant.

[43] Covic A, Haydar AA, Bhamra-Ariza P, Gusbeth-Tatomir P, Goldsmith DJ (2005). Aortic pulse wave velocity and arterial wave reflections predict the extent and severity of coronary artery disease in chronic kidney disease patients.. J Nephrol.

[44] Wiggins KJ, Rumpsfeld M, Hawley CM, O'Shea A, Isbel NM (2007). Baseline and time-averaged fluid removal affect technique survival in peritoneal dialysis in a non-linear fashion.. Nephrology (Carlton).

[45] Davies SJ, Brown EA, Reigel W, Clutterbuck E, Heimburger O (2006). What is the link between poor ultrafiltration and increased mortality in anuric patients on automated peritoneal dialysis? Analysis of data from EAPOS.. Perit Dial Int.

[46] van Biesen W, Heimburger O, Krediet R, Rippe B, Lamilia V (2010). Evaluation of peritoneal membrane characteristics: a clinical advice for prescription management by the ERBP working group.. Nephrol Dial Transplant.

[47] Rodriguez-Carmona A, Fontan MP (2002). Sodium removal in patients undergoing CAPD and automated peritoneal dialysis.. Perit Dial Int.

[48] Johnson DW, Hawley CM, McDonald SP, Brown FG, Rosman JB (2010). Superior survival of high transporters treated with automated versus continuous ambulatory peritoneal dialysis.. Nephrol Dial Transplant,.

[49] Davies SJ (2006). Overfill or ultrafiltration? We need to be clear.. Perit Dial Int.

